# A Hemodialysis Patient with Severe COVID-19 Pneumonia

**DOI:** 10.7759/cureus.7995

**Published:** 2020-05-06

**Authors:** Adel A Alalwan, Abdulraqeeb Taher, Ali H Alaradi

**Affiliations:** 1 Nephrology, Salmaniya Medical Complex, Manama, BHR

**Keywords:** hemodialysis, covid-19, coronavirus

## Abstract

Coronavirus disease 2019 (COVID-19) is an infectious disease caused by a novel coronavirus that has spread rapidly, resulting in a worldwide pandemic. Even though end-stage renal disease (ESRD) patients are particularly susceptible to COVID-19 infection and can develop severe to critical disease, there are limited studies and case reports about COVID-19 in ESRD patients. We report a case of a 63-year-old gentleman with ESRD on regular hemodialysis. We describe the clinical presentation of this patient, the diagnostic process, the laboratory and imaging investigations, as well as the course of treatment. He positively responded to a 14-day course of Lopinavir-Ritonavir, Ribavirin, Azithromycin, and Hydroxychloroquine.

## Introduction

In December 2019, a novel coronavirus was recognized as the cause of a group of pneumonia cases in Wuhan, a city in the Hubei Province of China. It quickly spread, resulting in an epidemic throughout China, followed by a worldwide pandemic with almost 2 million confirmed cases. In February 2020, the World Health Organization (WHO) named the disease COVID-19, which stands for coronavirus disease 2019. The virus that causes COVID-19 was named severe acute respiratory syndrome coronavirus 2 (SARS-CoV-2) [1].

Although severe COVID-19 disease can occur in otherwise healthy individuals of any age, it predominantly affects adults with advanced age or underlying medical co-morbidities [2, 3]. End-stage renal disease (ESRD) is a severe medical condition with a high prevalence of co-morbid conditions including diabetes, hypertension, and cardiovascular disease [4]. Even though ESRD patients are particularly susceptible to COVID-19 infection and can develop severe to critical disease, there are limited studies and case reports about COVID-19 in ESRD patients.

We report a case of an ESRD patient on regular hemodialysis with severe COVID-19 pneumonia. This report describes the clinical presentation of this disease in a hemodialysis patient, the diagnostic process, the laboratory and imaging investigations, as well as the course of treatment.

## Case presentation

A 63-year-old hemodialysis patient presented with a history of productive cough with whitish sputum and worsening shortness of breath for four days duration. His shortness of breath was not associated with orthopnea or paroxysmal nocturnal dyspnea. His wife was confirmed to have COVID-19 disease three days before his symptoms started. He denied any history of fever, sore throat, nasal congestion, or headache. He was later admitted in a special isolation hospital designated for COVID-19/ suspected COVID-19 patients in Bahrain. He has a medical history of hypertension, diabetes mellitus type 2, ischemic heart disease, and ESRD. He has been on hemodialysis three-times-weekly through a tunneled vascular catheter. His regular medications include Sevelamer, Calcitriol, Rosuvastatin, Amlodipine, and Perindopril. His blood sugar is controlled without medications. 

Based on the Ministry of Health in Bahrain guidelines, the hemodialysis facility where the patient was receiving his scheduled dialysis took specific precautions to reduce transmission and protect the patients. The facility was disinfected and the patient’s close contacts (including healthcare staff and other patients) were traced, tested for the virus, and asked to self-isolate for 14 days [5]. All his close contacts tested negative.

On admission, the patient was vitally stable, afebrile and was maintaining an oxygen saturation of 85% on room air. Although he was able to lie flat, he had labored breathing and his respiratory rate was approximately 25 breaths per minute. He was kept on a face mask with an oxygen flow of 6-7 L / min (PaO_2_ / FiO_2_ ratio of 322). On chest examination, he had symmetrical breath sounds bilaterally, with coarse inspiratory crackles that were more prominent on the right side. His cardiovascular examination showed normal heart sounds with no murmurs or added sounds and his jugular venous pressure was not raised. He did not have peripheral edema. His abdominal and neurological examinations were unremarkable.

Laboratory tests on admission showed a white blood cell count of 3.23 X 10^9^ per L with 62% neutrophils, 28.3% lymphocyte (absolute lymphocyte count 818.72 cells/ µL), and 2.2% eosinophils. His hemoglobin was 8.3 g/dl and his platelet count was 113 X 10^9^ per L. His urea was 18.4 mmol/L and his creatinine was 716 µmol /L. His sodium was 138 mmol/L, potassium 4.7 mmol/L , chloride 99 mmol/L, bicarbonate 26 mmol/L, calcium 2.01 mmol/L, phosphorus 2 mmol/L and magnesium 0.8 mmol/L. C-reactive protein (CRP) and procalcitonin (PCT) were 20.9 mg/L (Reference range 0-3 mg/L) and 0.58 µg /L (reference range 0-5 µg /L) respectively. His ESR was 45 mm/hour, Serum ferritin 2070 µg /L, and D-dimer 22.12 mg/L. His liver function tests and cardiac enzymes were within the normal range.

**Figure 1:**
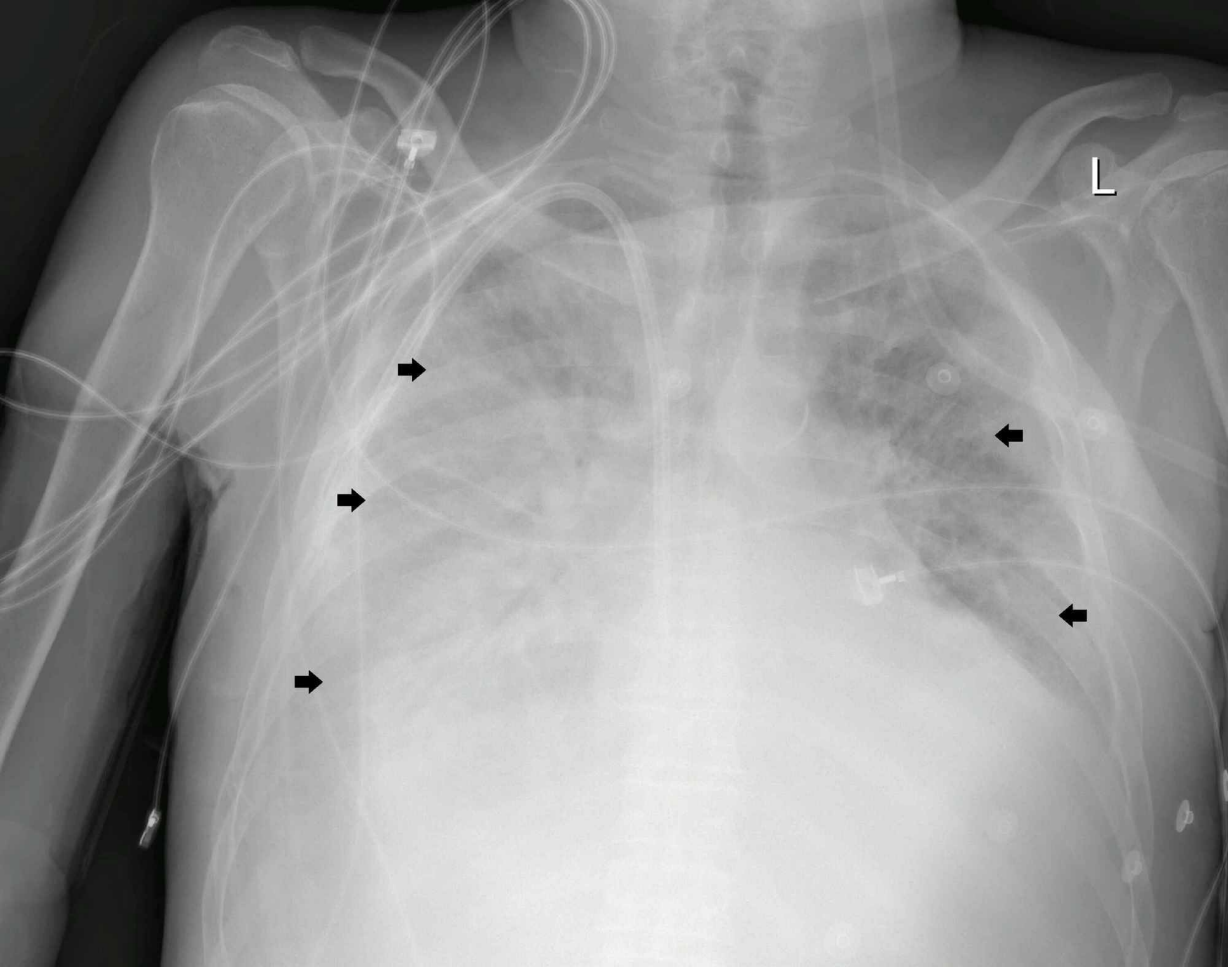
portable chest x-ray (24 hours after admission) showing bilateral patchy lower lobe predominant airspace opacification (Black Arrows).

Approximately 24 hours after admission, the patient was shifted to an isolated high dependency unit for additional monitoring after further desaturation despite oxygen therapy (PaO_2_ / FiO_2_ ratio 250). An urgent portable chest x-ray revealed bilateral patchy lower lobe predominant airspace opacification more diffusely involving the right lung, and more peripherally in the left lung (Figure 1).

A nasopharyngeal swab was taken from the patient and it was positive for SARS-CoV-2 virus nucleic acid. In addition, a septic workup was sent to rule out any bacterial co-infection.

The patient was diagnosed with severe COVID-19 disease. He was started on an antibiotic regimen designed for COVID-19 pneumonia as well as empiric antibiotic therapy for possible co-infection. He received a 14-day course of Lopinavir-Ritonavir, Ribavirin, Azithromycin, and Hydroxychloroquine. He also finished a 10-day course of Vancomycin, Meropenem, and a 5-day course of Oseltamivir. The antibiotic dosages have been adjusted for ESRD and hemodialysis. He was kept on deep venous thrombosis prophylaxis with heparin subcutaneous injections and received his regular medications.

An electrocardiogram (ECG) was done every alternate day to monitor the QT interval on antibiotic therapy which did not exceed 450 msec. His ECG showed baseline findings including a first-degree Atrioventricular block with poor R-wave progression, and no acute ischemic changes were noted. His repeated echocardiogram on this admission did not reveal any new findings. He had a left ventricular ejection fraction of 35 -40% with persistent regional wall motion abnormalities noted in septal and apical regions. His central and peripheral blood cultures were sterile on two separate occasions. He also had a sterile endotracheal aspirate culture.

The patient responded gradually to medical therapy and his oxygenation continued to improve until he was successfully weaned from oxygen. He continued to receive his hemodialysis sessions as scheduled bedside. His fluid balance was monitored closely, and adequate ultrafiltration was ensured. His blood count, electrolytes, and ECG findings remained consistent during the hospital stay.

**Figure 2:**
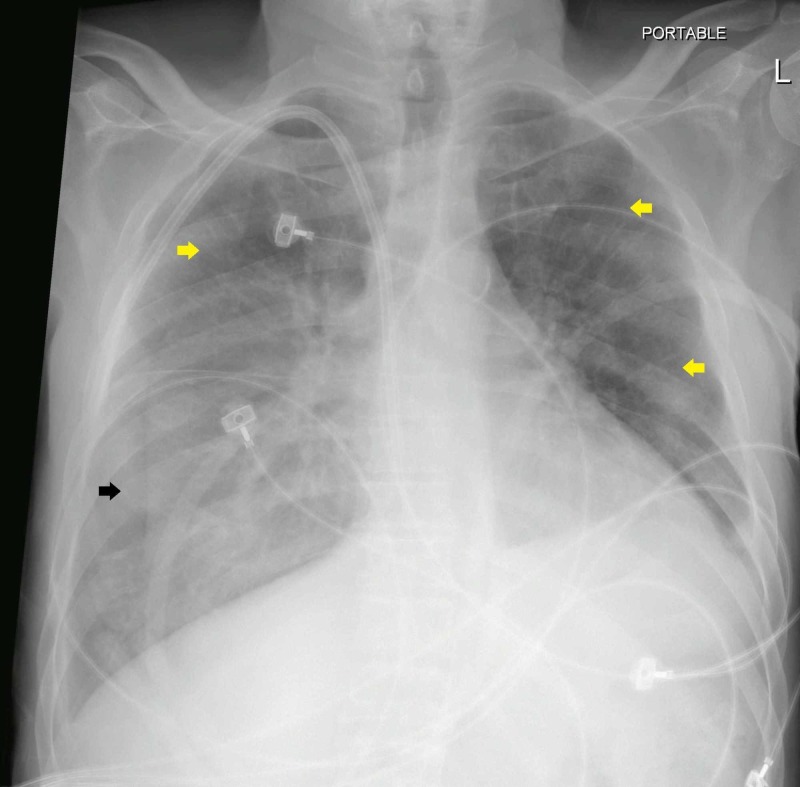
Portable chest x-ray (14 days after admission) showing partial interval resolution of the opacities in the left lung and upper zone of the right lung (Yellow Arrows). Remaining opacities are visible in the right lower lobe (Black Arrow).

After 14 days in the high dependency unit, his condition improved clinically, and he was shifted out to the ward under isolation settings. A repeated SARS-CoV-2 virus nucleic acid swab was negative. Although the patient's radiographic findings lagged behind the improvement in clinical condition, his repeated chest x-ray showed partial interval resolution of the opacities which was more evident in the left lung and upper zone of the right lung (Figure 2).

## Discussion

COVID-19 is an infectious disease that is mainly transmitted from person to person via respiratory droplets. COVID-19 pneumonia appears to be the most common serious manifestation of the disease. It is predominately characterized by fever, cough, dyspnea, and bilateral infiltrates on chest imaging [6, 7]. Atypical presentations of COVID-19 disease are frequent in hemodialysis patients and they are usually difficult to distinguish from other symptoms common among these patients. Wang et al. reported five cases of COVID-19 disease in hemodialysis patients in Zhongnan Hospital of Wuhan University. They found that only three out of five had a fever and that the typical triad of fever, cough, and dyspnea was not present in any of the patients reported [8]. In our case, the patient did not have any documented fever spikes on admission or during the hospital stay.

The patient was diagnosed based on the Ministry of Health in Bahrain guidance for COVID-19 case definitions and the Chinese Clinical Guidance for COVID-19 Pneumonia Diagnosis and Treatment (Trial version 7). He was labeled as suspected case based on having close contact with a confirmed COVID-19 case and presenting with respiratory symptoms, imaging features suggestive of COVID-19 pneumonia as well as lymphopenia on admission. The diagnosis was confirmed by identifying SARS-CoV-2 virus nucleic acid by Real Time Reverse-Transcription Polymerase Chain Reaction (RT-PCR). The case was further classified as a severe disease due to having an oxygen saturation below 93% at rest on admission and having more than 50% lung involvement on imaging within 24 hours [5, 9].

Our patient exhibited laboratory findings common among COVID-19 patients [7, 9]. He had lymphopenia as well as raised inflammatory markers (ESR, CRP, Serum ferritin, D-dimer). Although the majority of COVID-19 pneumonia patients have normal serum procalcitonin (PCT), the patient had slightly elevated PCT on admission. Tang et al. noted that PCT has limitations in hemodialysis patients with COVID-19 pneumonia because it tends to be chronically elevated in these patients [10].

Common abnormal radiographic findings in the chest x-rays of COVID-19 pneumonia patients were consolidation and ground-glass opacities, with bilateral, peripheral, and lower lung zone distributions. Our patient had typical chest x-ray findings (Figure 1); thus CT-scan was not needed for diagnosis [11].

The patient management plan was based on evidence-based medicine as well as the protocols published by the Ministry of Health in Bahrain for the treatment of severe COVID-19 pneumonia. The COVID-19 treatment approach remains uncertain with no therapies clearly proven effective. In addition to supportive care, the patient was started on a COVID-19 regimen composed of Lopinavir-Ritonavir, Ribavirin, Azithromycin, and Hydroxychloroquine [5]. The dosages of the administered therapy were adjusted for ESRD and hemodialysis to ensure their safety and efficacy. The QT interval was also closely monitored in our patient because ESRD patients are at high risk for developing QT interval prolongation and life-threatening arrhythmias with the COVID-19 regimen. They are particularly vulnerable due to their susceptibility to electrolyte disturbances such as hypocalcemia and hypomagnesemia.

The evidence on the possible efficacy of the proposed regimen for severe COVID-19 pneumonia is largely from case reports and observational studies. Lopinavir-ritonavir and Ribavirin have been used successfully as monotherapies in the treatment of mild COVID-19 pneumonia in hemodialysis patients [8, 10]. In 2004, Chu et al. noted a favorable clinical response with lopinavir/ritonavir and ribavirin combination in 41 SARS‐CoV patients when compared with the historical outcomes of ribavirin and corticosteroids [12]. The combination of Azithromycin and Hydroxychloroquine was also suggested to be associated with a more rapid resolution of virus detection compared with Hydroxychloroquine alone [13].

Empiric antibiotic therapy for possible co-infection was also initiated. The choice of empiric antibiotics was based on clinical judgment, the bacterial pathogens commonly isolated at the patient’s hemodialysis facility, and his own bacterial culture history. Oseltamivir was added to empiric therapy because influenza pneumonia was included in the initial differential diagnosis based on clinical presentation and chest x-ray findings.

After discharging the patient, he will be in home quarantine for another 14 days. He will be tested again for the virus at day 7 and day 14 post-discharge. He will also be monitored closely for any respiratory and gastrointestinal symptoms. Meanwhile, precautions will be taken to isolate the patient in the dialysis facility and minimize his contact with the healthcare staff. Safety measures will also be taken in transporting the patient to and from the dialysis facility.

## Conclusions

ESRD patients with COVID-19 can present atypically and the index of suspicion should be high in the setting of a pandemic to avoid exposure to healthcare staff and other hemodialysis patients. COVID-19 therapy in ESRD is especially challenging due to the requirement of renal dose adjustment of various medications and the propensity of ESRD patients to develop QT prolongation due to concomitant hypocalcemia and other electrolyte imbalances. Further clinical trials and observational studies are required to clearly understand the whole spectrum of clinical presentations and the optimal diagnostic and treatment methods for COVID-19 disease in hemodialysis patients.
